# Advancements in Gas Separation for Energy Applications: Exploring the Potential of Polymer Membranes with Intrinsic Microporosity (PIM)

**DOI:** 10.3390/membranes13120903

**Published:** 2023-12-06

**Authors:** Carmela Astorino, Eugenio De Nardo, Stefania Lettieri, Giuseppe Ferraro, Candido Fabrizio Pirri, Sergio Bocchini

**Affiliations:** 1 Center for Sustainable Future Technologies (CSFT), Istituto Italiano di Tecnologia (IIT), Via Livorno, 60, 10144 Torino, Italy; carmela.astorino@polito.it (C.A.); eugenio.denardo@polito.it (E.D.N.); fabrizio.pirri@polito.it (C.F.P.); 2 Department of Applied Science and Technology, Politecnico di Torino, Corso Duca Degli Abruzzi, 24, 10129 Torino, Italy; giuseppe.ferraro@polito.it

**Keywords:** polymers of intrinsic microporosity, CO_2_, gas separation, carbon capture, natural gas/biogas upgrading

## Abstract

Membrane-based Polymers of Intrinsic Microporosity (PIMs) are promising candidates for energy-efficient industrial gas separations, especially for the separation of carbon dioxide over methane (CO_2_/CH_4_) and carbon dioxide over nitrogen (CO_2_/N_2_) for natural gas/biogas upgrading and carbon capture from flue gases, respectively. Compared to other separation techniques, membrane separations offer potential energy and cost savings. Ultra-permeable PIM-based polymers are currently leading the trade-off between permeability and selectivity for gas separations, particularly in CO_2_/CH_4_ and CO_2_/N_2_. These membranes show a significant improvement in performance and fall within a linear correlation on benchmark Robeson plots, which are parallel to, but significantly above, the CO_2_/CH_4_ and CO_2_/N_2_ Robeson upper bounds. This improvement is expected to enhance the credibility of polymer membranes for CO_2_ separations and stimulate further research in polymer science and applied engineering to develop membrane systems for these CO_2_ separations, which are critical to energy and environmental sustainability. This review aims to highlight the state-of-the-art strategies employed to enhance gas separation performances in PIM-based membranes while also mitigating aging effects. These strategies include chemical post-modification, crosslinking, UV and thermal treatment of PIM, as well as the incorporation of nanofillers in the polymeric matrix.

## 1. Introduction

### 1.1. Background

In order to address global greenhouse gas (GHG) emissions [1], which are primarily driven by carbon dioxide (CO_2_) [2], and to achieve the long-term objectives outlined in the Paris Agreement [3] (Adoption of the Paris Agreement, FCCC/CP/2015/L.9/Rev.1, UNFCCC, 2015), Carbon Capture and Storage (CCS) and Carbon Capture and Utilization (CCU) and their combination as Carbon Capture Storage and Utilization (CCSU) have emerged as pivotal strategies for carbon mitigation [4].

In fact, over the past 10 years, atmospheric carbon dioxide concentration has risen by more than 20 ppm, averaging an increase of approximately 2 ppm annually, culminating in a level of 415 ppm in 2022 [5,6].

CCS focuses on the efficient separation of CO_2_ from specific point sources such as power plant flue gas (CO_2_/N_2_) [7], syngas in hydrogen production (CO_2_/H_2_), as well as natural gas (NG) and biogas (CO_2_/CH_4_) [8]. On the other hand, CCU not only seeks to reduce emissions released into the atmosphere but also leverages the potential of CO_2_ by integrating it into various industrial processes. This approach substitutes traditional raw materials and brings about added benefits [9].

Membrane technology can serve this dual purpose, as it can have high selectivity for CO_2_/CH_4_ and for CO_2_/N_2_ gas pairs. It not only aids in reducing atmospheric CO_2_ levels, such as emissions from power plants, but also bolsters the production of sustainable fuels like CH_4_ and biogas necessary also for the future scarcity, considering the necessity of fossil fuel substitution.

Biomethane, produced through biogas upgrading, stands out as a promising and sustainable renewable energy source. Numerous technologies, including cryogenic separation, chemical and physical absorption, water scrubbing, adsorption, and membrane separation, have been developed for biogas upgrading. Of these, in the past decade, membrane separation has gained investment costs ranging from 3500 to EUR 7500(m^3^ h^−1^) and operational costs of EUR 7.5–12.5(m^3^ h^−1^) [8]. When it comes to biogas upgrading and cost-effective carbon capture, polymeric materials have emerged as the preferred materials for membrane fabrication. More specifically, ultra-permeable Polymers with Intrinsic Microporosity (PIMs) have emerged as leading candidates, credited to their superior gas permeability while maintaining an impressive CO_2_ selectivity.

Past literature has already highlighted the potential of PIM-based polymers in gas separation applications [10], but the field still holds significant opportunities for future advancements. By building upon the existing research and continuously exploring and improving PIM-based materials, we can anticipate outcomes that are even more remarkable. Efforts to enhance the performance of these membranes through advanced polymer synthesis, novel modifications, and optimized membrane fabrication methods are likely to yield substantial improvements. The future prospects in this field are encouraging, as ongoing research and development can address the existing limitations of PIM-based membranes, such as selectivity, permeability, and long-term stability, enabling the design of membranes that are highly efficient in separating specific gas mixtures.

### 1.2. Advantages of Polymeric Membranes

Gas separation membrane classification often depends on the materials that constitute them. Generally, they fall into two primary categories: organic (polymeric) and inorganic, which include metallic and ceramic variations often derived from zeolites or microporous structures like Metal−Organic Frameworks (MOFs).

Metallic membranes, despite their impressive efficacy and durability, encounter limitations due to their reliance on high-cost metals such as platinum and palladium, coupled with their need for extremely elevated operational temperatures (200−900 °C). These combined drawbacks challenge their widespread adoption in the market.

In contrast, inorganic membranes constructed from zeolites or MOFs excel in their separation capabilities. However, they grapple with issues related to brittleness, high production costs, and constraints in processability and scalability [11,12]. These attributes, despite their advanced separation proficiency, limit their broad-scale commercial allure.

Organic (polymeric) membranes, conversely, benefit from their cost-efficient production materials and their capability to operate at significantly reduced temperatures. Their robust performance paired with commendable mechanical and chemical resilience [13] has positioned them favorably in a multitude of industries. Consequently, this review will predominantly spotlight polymeric membranes, reflecting their commercial prominence in gas separation and the current research direction.

### 1.3. Fabrication Methods

The efficacy of membrane performance is complex and multifaceted, transcending the mere inherent qualities of the polymer utilized. While the base attributes of the polymer are undoubtedly crucial, the overarching functionality of the membrane is sculpted by an array of factors. These encompass its distinct morphology, the methodologies adopted during fabrication, and the specifics of its preparation. The membrane’s structure, whether classified as asymmetric, symmetric, or composite, has a profound impact on its selectivity and permeability. Additional elements, like the conditions under which phase inversion occurs and the choice of solvents, can notably modify attributes like porosity, thickness, and surface behavior. Although the foundational aspects are rooted in the polymer’s characteristics, achieving pinnacle performance in a membrane is a delicate balance between its material properties and the intricacies of its fabrication.

A nuanced understanding of membrane design and production accentuates the need to appreciate the harmonious interplay among various factors, such as material choice, fabrication strategies, and preparation nuances. Such insights are invaluable for tailoring membranes to specific requirements, assuring their longevity and achieving optimal separation efficiencies. Prudent selection and design not only optimize performance but also foster economic viability, positioning membranes as indispensable tools in commercial ventures. This comprehensive perspective ensures membranes maintain their pivotal role in addressing global challenges in gas separation processes. However, this review places a significant emphasis on materials, sidelining the intricate details of membrane preparation. For those seeking an in-depth exploration of membrane preparation, recent reviews such as [14] are highly recommended.

### 1.4. General Outlook

To enhance membrane capabilities, there are several paths to explore: (i) employing materials more durable and resilient to mechanical, chemical, and thermal stresses; (ii) selectively increasing permeability and selectivity in areas where these enhancements would be most beneficial; and (iii) placing greater emphasis on understanding the fundamental relationships between membrane structures, properties, and processing.

In light of current demands, it is crucial to prioritize the development of membranes with superior selectivity rather than focusing solely on improving their permeability. In a polymer film, the presence of pores and channels of different sizes and structures results in a wide distribution of free-volume elements. As a consequence, the polymer’s capacity to achieve molecular separations is undermined. However, dense vitreous polymers have large free-volume structures that exhibit exceptional transport and separation abilities for molecules. These structures can surpass the limitations of traditional polymers. By inducing segment rearrangement, the microstructure of these materials can be intentionally modified. The degree of rearrangement, the original chain’s flexibility, and eventually the addition of small templating molecules can all be precisely controlled to customize the free-volume topology. This systematic approach to tailoring the architecture of free-volume elements offers a promising strategy for developing high-performance polymers capable of achieving molecular-scale separations.

## 2. Permeability-Selectivity Trade-Off

The commercialization of polymeric membranes, which began in the late 1970s, led to a comprehensive database detailing gas permeation and separation properties. This data accumulation provided the groundwork for Lloyd Robeson’s pivotal 1991 revelation. By plotting the data of known membranes on a Cartesian axis—with selectivity on the y-axis and permeability on the x-axis—Robeson analyzed their interrelationship [15,16,17]. He deduced an empirical understanding: to boost a membrane’s permeability, there would be a concomitant reduction in its selectivity and vice versa. As Robeson plotted each permeability and selectivity datum, a boundary line emerged, which he identified as an upper limit that contemporary membranes struggled to exceed. This trade-off suggests that the ability of polymers to separate gases in gas pairs is determined by their solubility and diffusion coefficient and in the end by permeability. Tighter spacing between polymer molecules reduces permeability but improves separation properties.

Freeman’s theoretical framework sheds light on the upper bounds of permselectivity for gases, underscoring the pivotal role of diffusivity in establishing the upper bound selectivity in polymer-based gas separations. Within this context, while solubility selectivity demonstrates marginal variations across different polymers, certain parameters remain steadfast for specific gas pair and polymer classifications. Empirical evidence supports this theory, suggesting that polymers with elevated glass transition temperatures and pronounced interchain spacing align closely with the upper bounds for several gas combinations. For enhanced polymeric membrane efficiency, it is essential to amplify solubility selectivity, bolster chain rigidity, and expand interchain distances [18,19].

With the stage set by this understanding, advancements in membrane architecture have zeroed in on pivotal attributes for next-gen membranes. These encompass the introduction of size-specific free-volume elements, a lean active layer, and meticulously calibrated interactions between gases and the membrane material—all contributing to enhanced gas selectivity. By adeptly navigating the equilibrium between permeability and selectivity, we are forging a path to more adept membranes suitable for diverse separation and refining procedures.

In the next part, PIMs will be discussed in this evolving landscape, especially in CO_2_/CH_4_ and CO_2_/N_2_ separations.

**Figure 1:**
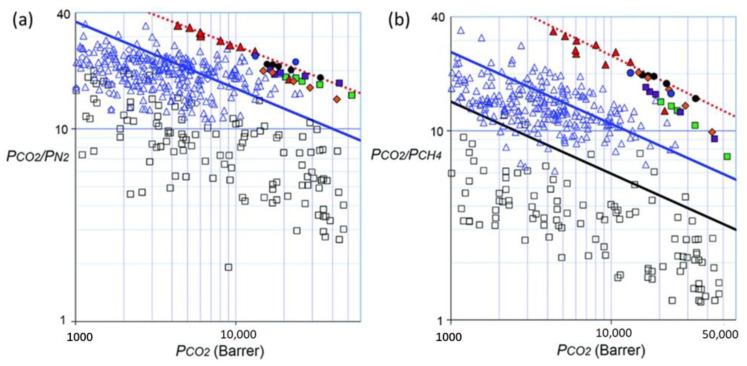
Robeson plots for the (**a**) CO_2_/N_2_ and (**b**) CO_2_/CH_4_ gas pairs. Previously reported data for non-PIM polymers (black square) and PIMs (blue triangle) are shown. Upper bounds are represented by black lines (1991) and blue lines (2008). The revised upper bounds for CO_2_/N_2_ and CO_2_/CH_4_ are shown as dotted red lines (red dotted square refers to experimental data for the revised upper bounds). The position of the gas permeability data for films of novel PIMs such as PIM with Benzotriptycene (PIM-BTrip, red triangle), PIM with hexamethylindane and triptycene (PIM-HMI-Trip, green square), PIM with dimethyl-Benzotriptycene (PIM-DM-BTrip, purple square), PIM with trifluoromethyl-Benzotriptycene (PIM-TFM-BTrip, blue circle), and PIM with trifluoromethyl-Benzotriptycene (PIM-DTFM-BTrip, black circle) are reported. Reproduced from [20] with permission from the Royal Society of Chemistry.

These membranes showcase substantial operational enhancements, aligning with the linear trends on Robeson’s renowned plots. Notably, they not only mirror but frequently surpass the recognized Robeson upper bounds for CO_2_/CH_4_ and CO_2_/N_2_ compared with non-PIM polymers (see Figure 1).

### 2.1. PIM Membranes, an Alternative in CO_2_/CH_4_ and CO_2_/N_2_ Separation

While PIMs are a promising class of materials for gas separation, it is important to compare their performance to other polymers that are already successfully used in this field. This will give us a better understanding of the relative advantages and disadvantages of PIMs and help us to identify areas where further research is needed.

In the past decade, many new membranes for CO_2_ separation have been developed and tested. Polyvinyl amine (PVAm) emerged as the most researched polymer, with diverse strategies employed to enhance CO_2_ transport properties, such as copolymerization and the inclusion of nano-sized particles [21]. Wang et al. and Deng et al. explored the integration of various nanoparticles into the PVAm matrix [22]. Yave et al. studied commercially available copolymers, like Pebax, for scalability [23]. Scofield et al. enhanced CO_2_ transport by adding polyethylene glycol (PEG) copolymers to a Pebax matrix [24]. Ionic liquids with polymers were researched for better CO_2_ separation performance [25]. High-temperature-facilitated transport membranes for CO_2_ capture were explored by Ho and team [26]. Chen et al. delved into the potential of Pebax coatings on porous PAN hollow fibers for CO_2_/N_2_ separation [27]. Hägg and colleagues extensively researched the possibility of post-combustion CO_2_ capture using fixed-site carrier membranes [28]. Membranes based on biomimetic material for CO_2_ hydration were developed by Yao et al. [29]. Oh et al. reported on a polyvinylpyrrolidone (PVP)/potassium fluoride electrolyte membrane for facilitated CO_2_ transport in 2013 [30]. The main challenge with facilitated transport membranes is their dependency on CO_2_ partial pressure; however, they remain promising for post-combustion CO_2_ capture.

In Table 1, some values of polymers/copolymers commercially available from the literature are presented. While the selectivity is comparable to PIMs (refer to Table 2 and Table 3 in the other sections), the permeabilities are an order of magnitude lower. This indicates that once issues related to aging and membrane production are addressed, PIMs could become the true market leaders in gas separation.

On the other hand, CO_2_/CH_4_ separation is pivotal for NG extraction, optimizing the value of sweetened gas and minimizing corrosion and pipeline volume. Since the introduction of membranes for NG processing in the 1980s for CO_2_ removal [33], the field has seen the predominant use of materials like polyimides, cellulose acetate, select perfluoropolymers, and polysulfone (PSF). These membranes generally exhibit a CO_2_ permeaof nce up to 100 GPU and a CO_2_/CH_4_ selectivity close to 20 in real industrial scenarios [34].

In the last decade, excluding PIMs, a plethora of membrane materials, both inorganic and polymeric, have emerged for lab-scale CO_2_/CH_4_ separation, including diverse polyimides [35,36,37] and thermally rearranged (TR) polymers [38]. Mixed matrix membranes (MMMs), incorporating a range of organic and inorganic particles, have emerged as innovative solutions in NG sweetening, as extensively discussed in recent literature [39,40]. This field has seen significant advancements, highlighted in a comprehensive review [41]. However, certain materials, such as thermally rearranged (TR) polymers and MMMs, present notable fabrication challenges. Specifically, in ternary MMMs, the use of liquid additives has been a key strategy to enhance polymer−nanoparticle compatibility. Yet, these additives are prone to leaching under high operating pressures, prompting a need for careful optimization of component ratios, improved storage conditions, and the application of crosslinking techniques to bolster membrane stability. To further refine these membranes, advanced characterization methods are vital for a thorough interface analysis and to elucidate the effects of functionalized additives on the pore size of fillers. In this context, the development of new filler materials, particularly Metal−Organic Frameworks (MOFs), is gaining attention. MOFs, known for their exceptional separation performance, require simpler synthesis processes and have improved compatibility with various additives for enhanced efficacy. Moreover, the complexity of interactions within ternary MMMs underscores the necessity for sophisticated performance analysis models. These models are instrumental in predicting membrane behavior, taking into account the multifaceted interplay between the filler, functionalized additives, and the polymer matrix. Lastly, the practical application of ternary MMMs hinges on their performance under real-world conditions. Rigorous testing in environments that simulate actual operational challenges, including the presence of impurities like SO_2_ and NOx and varying temperature, humidity, and pressure, is essential to validate their efficacy and durability in industrial settings.

### 2.2. PIMs

PIMs are a unique category of glassy polymers, distinct for their vast free volume. This feature arises from the polymers’ rigid and twisted macromolecular backbones, which prevent tight packing. PIM-1 stands as the prototype for this class, derived from step-growth polymerization, which involves nucleophilic aromatic substitution reactions [15,16]. According to the IUPAC definition, micropores are voids smaller than 2 nm. The term “intrinsic microporosity” describes a continuous network of interconnecting intermolecular voids that arises due to the shape and rigidity of the constituent macromolecules.

Since the introduction of PIMs as a class of microporous polymers for gas separation technology, the upper bond limits have been surpassed, underlying their potential. Notably, benzotriptycene-based PIMs have showcased exceptional capabilities, outperforming the current SoA for CO_2_ over CH_4_, (for upgrading of natural gas and biogas), and for CO_2_ over N_2_ (for carbon capture from power plants) separation technology [20]. The gas permeability and selectivity data for some of this series of PIMs fall into a linear correlation on the benchmark Robeson plots, being parallel to, but considerably above, that of the 2008 CO_2_/CH_4_ and CO_2_/N_2_ upper bounds.

However, one of the primary obstacles to the practical use of PIMs in gas separation membranes is the phenomenon known as “aging”. This refers to the gradual decrease in available free volume over time. This aging process is especially pronounced in polymeric thin films, where permeability can drop by over 50% in just a few days [42]. Strategies such as thermal crosslinking [43], nanofiller incorporation [44], and alcohol treatment followed by thermal treatment [45] have been effective in mitigating physical aging.

In the following sections, we will focus on several pivotal areas in the field of gas separation using PIMs. First, we will explore the enhanced performance of PIM-based membranes, delving into the latest advancements in chemical post-modification, crosslinking, and UV and thermal treatments to improve their gas separation efficiency. Additionally, we will address the critical issue of physical aging in PIM membranes, examining innovative strategies to mitigate these effects and enhance membrane stability. A significant portion of our discussion will be dedicated to the development and characterization of PIM-based MMMs, incorporating a variety of inorganic, organic, and metallic−organic nanofillers, to overcome the limitations of pure PIM membranes and enhance their practical applications. In all sections, we will focus on the application of these advanced polymers in CO_2_ separation processes, emphasizing their role in natural gas/biogas upgrading and carbon capture from flue gases. This aligns with global efforts towards environmental sustainability and the reduction of greenhouse gas emissions. Through these focused discussions, our paper aims to provide a comprehensive overview of the current state and future prospects of PIM-based membranes in energy-related gas separation applications.

## 3. Recent Strategies in PIM Modification

### 3.1. Novel PIMs

Since the introduction of the Robeson upper bond in 2008 [16], a number of PIMs with enhanced rigidity have been discovered that exhibited gas permselectivity levels exceeding the 2008-established upper bounds [46]. The primary approach involved replacing the more flexible spirobisindane structural unit with alternatives such as spirobifluorene units [47]. Other substitutions include highly rigid bridged bicyclic constituents like Trögers base [48,49], triptycene and benzo-triptycene [20,49,50,51,52], ethanoanthracene [48,53,54,55], and methanopentacene [56].

**Figure 2:**
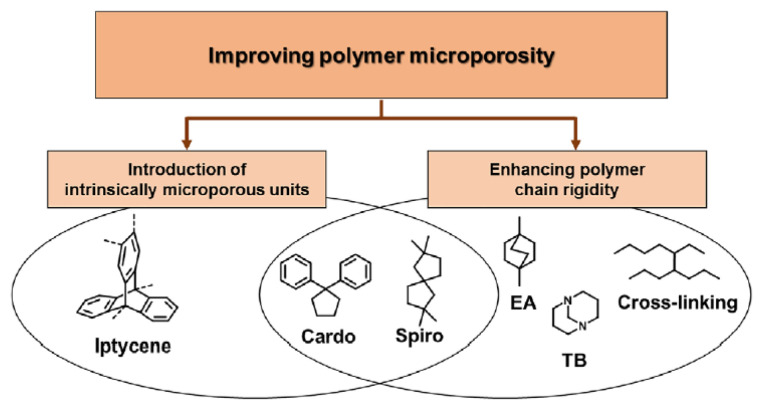
Strategies employed to enhance microporosity in polymeric membrane materials; these involve the integration of bulky and rigid moieties into the polymer backbone or through crosslinking processes. Reprinted with permission from [57]. Copyright © 2023 Wiley Periodicals, Inc.

Recent reviews have extensively covered advancements in novel PIMs, with notable mentions from 2020 [57] and 2021 [42]. These reviews underscore the recent progress in the design of tunable and finely engineered PIMs that challenge the Robeson upper bond. To maximize the gas transport properties of microporous polymers and enhance their gas separation performance, researchers can adjust the polymeric structure by (i) introducing intrinsically microporous units and/or (ii) improving chain rigidity to enhance microporosity in traditional polymers (see Figure 2).

**Figure 3:**
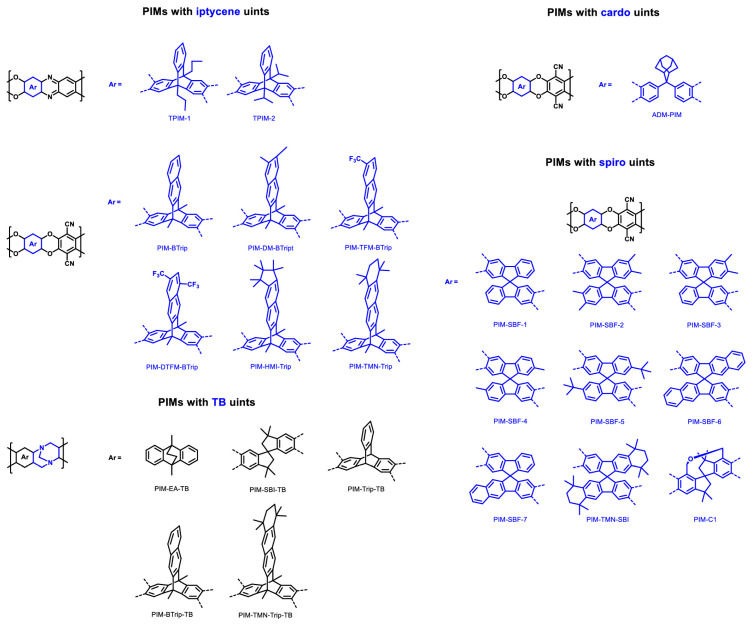
Recent advancements in PIM polymers showcasing enhanced microporosity and increased polymer rigidity. Reprinted with permission from [57]. Copyright © 2023 Wiley Periodicals, Inc.

In the past two decades, following the groundbreaking work of Budd and McKeown on PIM-1 [58,59], researchers have leveraged insights from prior investigations to craft novel synthetic monomers. These are characterized by the inclusion of bulky substituents, which are designed to impede effective polymer chain packing. Notable examples of such bulky structural functionalities can be seen in Figure 3. These include shape-persistent iptycenes such as triptycene, benzotriptycene, or pentiptycene. Owing to their three-dimensional [2,2,2]-ring configurations, these structures offer significant internal free volume, making them prime candidates for gas separation applications. Additionally, cardo (meaning “loop” or “hinge” in Latin) and spiro monomers are alternative types of alicyclic synthetic monomers. Ethanoanthracene (EA) units and Troger’s base (TB) units also constitute a widely used class of monomeric units, contributing to the development of a new generation of PIMs that showcase enhanced gas separation capabilities.

Figure 2 depicts recently reported PIM polymers that demonstrate both increased microporosity and enhanced polymer rigidity [57].

### 3.2. Post-Modification of PIMs

Post-modification of PIMs can be achieved through various methods, including chemical reactions, thermal treatments, thermo-oxidative modifications, and photo-oxidation via UV irradiation. The primary objectives of these modifications are to enhance gas separation efficiency, mitigate physical aging, and bolster thermal stability. These enhancements are achieved by increasing the polymer’s affinity for the target gas, stabilizing the polymer structure, and fine-tuning the pore size dimensions [42].

#### 3.2.1. Chemical Post-Modification

The polymer’s proficiency in sieving CO_2_ can be effectively enhanced through chemical post-functionalization, integrating specific groups such as tetrazole, triazine, amines, hydroxyls, and imidazole, all recognized for their strong affinity to CO_2_ [60]. This common approach based on solubility-controlled chemical functionalization is a staple for acid-gas removal. By incorporating carriers capable of adsorbing the target gas and forming complexes, the solution−diffusion mechanism is harnessed to bolster selectivity [61]. For CO_2_ removal, carriers embedded with basic groups like amines and carboxylates prove optimal. These amines can further be categorized into primary, secondary, or tertiary.

**Figure 4:**
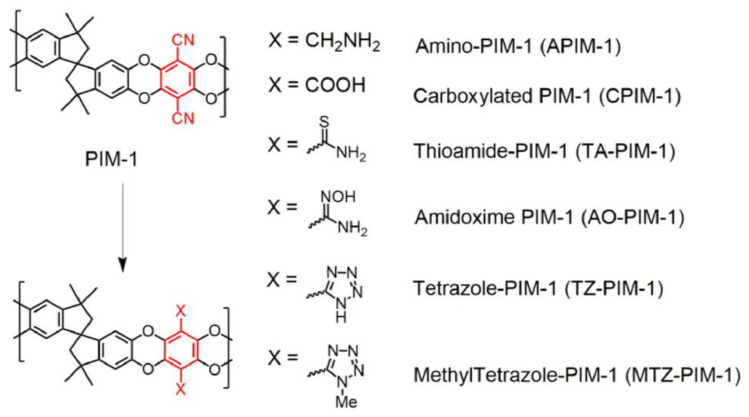
Various chemical post-modifications of PIM-1 utilized in the fabrication of gas separation membranes. Reprinted with permission from [44]. Copyright ©2021 Elsevier Ltd. All rights reserved.

The nitrile (−C≡N) groups in PIM-1 are promising functional sites for incorporating various CO_2_-philic groups (Figure 4). Nevertheless, many of these modifications often lead to decreased surface area or available cavities within the material [62]. Some of the techniques employed include carboxylate-functionalized PIM-1 (cPIM) [63,64,65,66], amidoxime-functionalized PIM-1 [62,67], heterocyclic tetrazole-functionalized PIM-1 [68], thioamide-functionalized PIM-1 [69] and amine-functionalized PIM-1 [70,71,72,73].

##### Carboxylate-Functionalized PIM-1 (cPIM-1)

In 2009, Du et al. pioneered the synthesis of carboxylated PIM (cPIM) materials, utilizing the alkaline hydrolysis of the nitrile group in PIM-1 [64]. The resulting polymer exhibited a notable improvement in permselectivity while displaying reduced permeability compared to the materialist precursor. In 2014, Satilmis et al. highlighted that the PIM-1 materials subjected to base hydrolysis (120 °C using 20% NaOH and at 100 °C with 10% NaOH in a water−ethanol mixture) consisted of a combination of various functional groups, including amide, carboxylic acid, ammonium carboxylate, and sodium carboxylate. Until then, PIM-1 hydrolysis was assumed to exclusively yield carboxylated products. Interestingly, the proportion of carboxylic acid groups was found to be less than 20% in these materials. Despite the use of an extensive base hydrolysis process, only a modest 51% carboxylation level was achieved in the final products [66].

In 2017, Jeon et al. successfully prepared a highly carboxylate-functionalized PIM (HCPIM) with approximately 92 mol% of carboxylic acid groups through a prolonged alkaline hydrolysis process lasting 360 h [63]. These HCPIMs were soluble in several organic solvents like tetrahydrofuran and dimethyl sulfoxide. Membranes of HCPIMs, crafted using the standard solution casting method, displayed closer interchain distances and enhanced affinity for CO_2_, highlighting their promise for selective gas separation and related applications.

Recently, a novel approach to synthesize carboxylate-rich PIMs (c-PIMs) from PIM-CONH_2_ using nitrous acid emerged [65]. Comprehensive characterization verified the full conversion of amide to carboxylic acid groups in c-PIM. While this c-PIM had reduced thermal stability compared to PIM-CONH_2_, it was soluble in diverse organic solvents, such as THF, acetone, DMSO, and NMP. Interestingly, some solvents prompted a side nitration reaction in the aromatic structures.

##### Amidoxime-Functionalized PIMs (AO-PIM)

Another effective strategy to enhance the polymer’s interaction with CO_2_ involves introducing hydroxyl- or amine-based moieties [60]. This can be easily achieved by modifying the nitrile groups. Prior studies have reported the successful functionalization of PIM-1 with amidoxime groups (AO-PIM), accomplished through a rapid nitrile conversion using hydroxylamine in THF at reflux temperature [62,67]. The incorporation of amidoxime functionality in PIM-1 not only increases CO_2_ capacity by up to 17% but also enhances the micropore surface area by 20% without compromising its film-forming ability [62]. Moreover, AO-PIM-1 exhibits a remarkable three-fold increase in αD(CO_2_/CH_4_) compared to PIM-1, surpassing the 2008 upper bound with P(CO_2_) = 1153 Barrer and ideal α(CO_2_/CH_4_) = 34. The presence of oxygen and nitrogen from the −OH and −NH_2_ groups, along with the dioxane rings acting as acceptors, creates a stable network through hydrogen bonding. While the introduced −OH and −NH_2_ groups enhance the polymer’s affinity towards CO_2_, they may lead to a reduction in accessible cavities, resulting in lower gas solubility, especially for CO_2_.

Miles et al. described another example of amidoxime-functionalized PIMs [70], utilizing a post-synthetic functionalization approach to introduce alkylamine functionalities. These sorbents not only exhibit the highest CO_2_ uptake among reported PIMs but also showcase the advantages of being tunable, processable, and stable in their polymeric sorbent design. As a result, they offer the potential for nanoengineering to address various gas capture and separation applications beyond CO_2_. The hydroxyl group is another CO_2_-philic group. Its post-synthesis introduction can therefore enhance CO_2_ selectivity. An example was reported in 2021 by Gao et al., where a composite membrane was fabricated by coating tannic acid, a polyphenol, onto the surface of PIM-1 using a simple dipping method [71]. Tannic acid contains numerous polar oxygen-containing groups, such as quinone and phenolic hydroxyl, which underwent self-polymerization on the membrane surface, creating a CO_2_-philic, defect-free, and thin layer. After the tannic acid coating, the resulting composite membranes showed an increase in CO_2_/CH_4_ selectivity while maintaining comparable or even higher gas permeability compared to the pristine PIM-1 membrane. The improved CO_2_/CH_4_ selectivity exceeded the reported 2008 upper bound, demonstrating the effectiveness of tannic acid as a coating material to enhance the gas separation performance of PIM-1 membranes.

##### Heterocyclic Tetrazole-Functionalized PIMs

Heterocyclic tetrazole rings can be introduced to PIM-1 using click chemistry reactions to enhance its selectivity of CO_2_ over other gases [68]. Initially, nitrile-containing PIM-1 undergoes a [2 + 3] cycloaddition reaction, leading to the formation of a polymer substituted with tetrazole groups (TZ-PIM). However, subsequent methylation produces a new polymer, where methyl tetrazole groups are introduced (MTZ−PIM). The MTZ−PIMs exhibited significantly enhanced CO_2_ solubility. Compared to PIM-1, MTZ−PIMs showcase superior gas permselectivity but reduced gas permeability for pure gas pairs like O_2_, N_2_, and CO_2_, as well as for mixed gases like CO_2_/N_2_. The data on selectivity, along with high gas permeability, closely approached the Robeson 2008 upper-bound performance limit for the O_2_/N_2_ and CO_2_/N_2_ pure gas pairs and even exceeded the upper-bound for the CO_2_/N_2_ mixed gas pair.

##### Amine-Functionalized PIMs

An alternative post-modification approach entails the conversion of PIM-1’s nitrile groups into primary amines, for example, borane complexes [72]. Introducing −NH_2_ groups augments CO_2_ uptake and enhances solubility/selectivity. However, it simultaneously promotes hydrogen bond formation between the amine hydrogens and oxygen in the dioxane rings, thus diminishing the polymer’s free volume. This strong interaction among the polymer chains reduces pathways for gas transport, resulting in a decrease in CO_2_ permeability and diffusivity. While unmodified PIM-1 favors CO_2_ in the H_2_/CO_2_ gas pair, amine−PIM-1 exhibits permselectivity towards H_2_. The combined reduction in both permeability and selectivity of amine−PIM-1 confirms its limited efficiency in surpassing the upper bounds.

Recently, Chen et al. introduced a facile, controllable, and versatile chemical vapor amination strategy, which simultaneously modifies gas sorption and diffusion properties in PIM-based membranes, resulting in enhanced carbon capture performance [73]. The method involved exposing the membranes to amine vapors under mild conditions, leading to nucleophilic substitution reactions between amines and ether/halogen groups. Such induced grafting by ring-opening, terminal replacement, or chain-scission/crosslinking of PIM-1 induces CO_2_-philicity and passageways within the membrane structure. The CO_2_-philic membranes prepared through this post-synthesis amination process demonstrated significantly enhanced CO_2_/N_2_ selectivity, surpassing 30.8, which corresponds to a remarkable 226% improvement compared to the original membrane. Additionally, these membranes maintain a high CO_2_ permeability of 2590 Barrer, allowing them to surpass the trade-off upper bound of conventional polymer membranes. These outcomes firmly position post-synthesis amination as a robust method for generating membranes with top-tier carbon capture competencies.

Competitive sorption is another mechanism that can be leveraged to enhance gas separation performance, leading to mixed-gas permselectivities that surpass pure-gas predictions [74]. To this end, the high-pressure and mixed-gas transport properties of six PIMs, all featuring identical benzodioxane backbones but differing in backbone functionalities, were investigated [74]. These functionalities included nitrile (−CN), carboxylic acid (−COOH), amine (−CH_2_NH_2_), tert-butoxycarbonyl (−CH_2_-NHCOOC(CH_3_)_3_), and partial urea (−NHCONH−). Notably, the amine-functionalized PIM-1 (PIM-NH_2_) exhibited unprecedented enhancements, with a 140% rise in equimolar CO_2_/CH_4_ mixed-gas permselectivity and a remarkable 250% boost in CO_2_/N_2_ mixed-gas permselectivity against pure-gas tests at 2 atm.

Furthermore, the incorporation of alkylamines into PIMs using acid−base and hydrogen-bonding interactions was also reported [75]. The incorporation of alkylamines into PIMs resulted in notable improvements, with a nearly four-fold increase in CO_2_ loading capacity and a significantly higher CO_2_/N_2_ selectivity over unmodified PIM-1. Moreover, these amine-appended PIMs demonstrated remarkable stability during adsorption/desorption isotherm cycles in both dry and humid conditions, typical of post-combustion CO_2_ capture processes.

##### Thioamide-Functionalized PIMs

Using phosphorus pentasulfide and sodium sulfite in a dioxane/ethanol mixture, nitrile groups in PIMs can be converted to thioamide functionalities [69]. However, this modification results in a significant 66% decrease in the surface area of the modified polymers if compared to the original PIM-1. This reduction is attributed to the bulkier side-chain moiety and the presence of hydrogen bonding between –NH_2_ donor groups and S and O acceptors, which leads to decreased inter-chain distances and reduced free volume. Interestingly, thioamide−PIM-1, when processed with ethanol, has been reported to exhibit a CO_2_ permeability of 1150 Barrer and a CO_2_/N_2_ selectivity of 30.3 [69].

#### 3.2.2. Crosslinking

**Figure 5:**
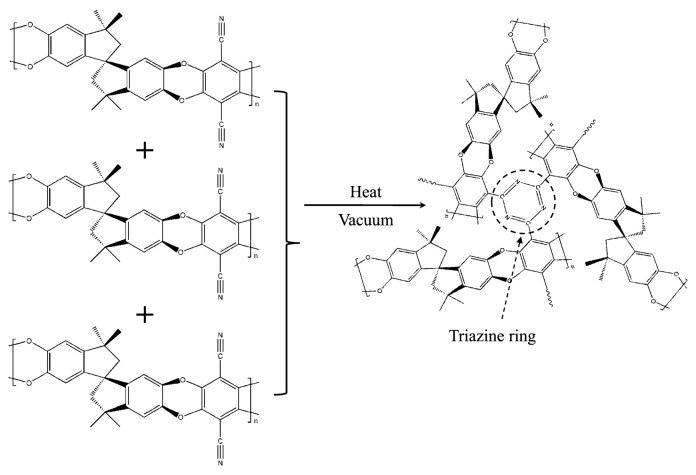
PIM-1 crosslinking through the thermally induced trimerization of nitriles into stable triazine rings, as commonly described in the literature. No release of volatile side products is observed. Reprinted with permission from [45]. Copyright © 2023, American Chemical Society.

Crosslinking represents an established method to create highly selective membranes for gas separation. By modulating the degree of crosslinking, it is possible to manipulate polymer solubility and precisely adjust membrane gas transport characteristics. Furthermore, intensifying the crosslinking degree yields superior thermal stability, achieved via elevated reaction temperatures and prolonged reaction periods. For example, PIM-1 thermally crosslinked by trimerization of cyanide to form triazine (Figure 5), under vacuum conditions at 300 °C for a 2-day reaction time, significantly enhances permselectivity properties.

The subsequent membrane showcases an 18% surge in CO_2_ permeability, a remarkable 104% improvement in CO_2_/N_2_ selectivity, and a notable 230% boost in CO_2_/CH_4_ selectivity [45]. Prolonging the crosslinking duration led to a more abundant formation of a greater number of triazine rings, which subsequently amplified the membrane performance efficacy. To be precise, the CO_2_ permeability soared to 4000 Barrer, CO_2_/N_2_ selectivity hit 41.7, and CO_2_/CH_4_ selectivity climbed to 54.8. These improvements are directly tied to the enriched presence of triazine rings, underscoring the pivotal role of the crosslinking duration in refining membrane attributes for optimized gas/gas separation outcomes [45].

When thermal crosslinking is conducted under an inert atmosphere with small amounts of oxygen, the formation of covalently crosslinked polymer chains through radical reactions was observed, leading to enhanced molecular sieving capabilities. The extent of crosslinking can be modulated by varying the oxygen content, reaction duration, and temperature [76]. Under conditions with elevated oxygen concentration, such as in air, the reaction duration needs to be restricted to roughly 10 min to prevent membrane degradation or cracking. An alternative methodology to craft crosslinked membranes involves converting the nitrile groups in PIM-1 into carboxyl groups. These are then eliminated by decarboxylation with annealing at 375 °C for 40 min [77]. The resulting phenyl radicals lead to biphenyl crosslinking. This crosslinking process enhances the selectivity of the resulting membranes for CO_2_/N_2_ and CO_2_/CH_4_ separation but also results in decreased CO_2_ permeabilities compared to the original, unmodified PIM-1 [77].

Another reported methodology involved cross-linking azide-modified polymers using nitrene reactions at diverse temperatures ranging from 150 to 300 °C [78]. The investigation indicated that azide groups within the repeating units decompose during thermal processes, prompting the release of nitrogen and leading to the creation of robust PIM networks. This resulted in a surge in gas permeability while almost preserving the initial selectivity. Variations in the spatial distribution and concentration of azide groups affected solubility and diffusion coefficients, which in turn impacted the development of free volume elements within the modified PIMs. An extensive aging study spanning about five months allowed for the evaluation of physical aging rates, correlating them to the concentration and distribution of the cross-linked azide groups. Findings underlined that heightened functionalization produced a pronounced sieving effect, especially for minuscule molecules like H_2_, consequently boosting gas separation selectivity.

Recently, Zhou et al. highlighted the development of hyper-cross-linked PIMs that showcase outstanding CO_2_ absorption capabilities, coupled with superb CO_2_/N_2_ and CO_2_/CH_4_ selectivity. This makes them strong contenders for carbon capture and biogas upgrading [79]. The starting hydrocarbon polymer backbones underwent strategic functionalization, integrating groups like −NO_2_, −NH_2_, and −SO_3_H, with the goal of refining their adsorption selectivity to preferentially favor CO_2_ over N_2_ and CH_4_. Even though this functionalization led to a decrease in the overall porosity, the final polymers exhibited enhanced CO_2_ uptake and selectivity against the target gases. Among these modified polymers, the sulfonated version demonstrated the highest CO_2_ uptake, achieving up to 298 mg g^−1^ (6.77 mmol g^−1^). In contrast, the aminated polymers showed the best CO_2_/N_2_ selectivity, achieving figures as high as 26.5. When considering CH_4_ separation, these aminated PIMs also stood out for their superior CO_2_ selectivity, registering values of up to 8.6. The observed advancements are the result of a coordinated combination of elements, including porosity, functional group selection, and the ideal isosteric heat of adsorption for the materials.

### 3.3. UV and Thermal Treatment

#### 3.3.1. Thermally Rearranged

Recent research indicates that it is possible to exceed the upper bound by utilizing microporous materials in membrane design. This includes PIMs and thermally rearranged (TR) polymers. These TR membranes provide a compelling blend of high permeabilities, selectivity, and excellent plasticization resistances, making them ideal for CO_2_ separation [44] (see Table 2). Effective enhancement of diffusion pathways can be achieved by controlling pore size distribution and free volume. Li et al. investigated the use of a hydroxyl-containing PIM-polyimide to develop a TR membrane [80].

**Table 2:** Performance of UV and thermally rearranged PIM membranes for CO_2_ separation.

Membrane	CO_2_ Permeability (Barrer)	CO_2_/N_2_ Selectivity	CO_2/_CH_4_ Selectivity	Ref.
PIM-1 ref. [45]	3375 (@3.5 Bar, 35 °C)	20.4	16.6	[45]
PIM-1 soaked in MeOH ref. [45]	6957 (@3.5 Bar, 35 °C)	20.7	14.8	[45]
PIM-300−2.0 d (Thermally cross-linked)	4000 (@3.5 Bar, 35 °C)	41.7	54.8	[45]
Untreated PIM	3934 (@1 Bar, 25 °C)	14.6	11.0	[63]
PIM-COOH-360 h	96.43 (@1 Bar, 25 °C)	53.6	25.2	[63]
TRIP-TR-400-30	840 (@2 Bar, 35 °C)	-	21	[81]
TRIP-TR-460-30 aged 85 days	444 (@2 Bar, 35 °C)	-	23	[81]
6FDA-DAT1-OH	43 (@2 Bar, 35 °C)	-	52	[81]
Untreated PIM ref. [82]	6601 (@3.5 Bar, 35 °C)	-	15.3	[83]
PIM-UV 30 min	724 (@3.5 Bar, 35 °C)	-	31.3	[83]
Untreated PIM ref. [84]	5622 (@4 Bar, 22 °C)	17.3	13.5	[82]
PIM-1, UV in air, 5 min	6007 (@4 Bar, 22 °C)	20.9	15.8	[82]
PIM-1, UV in air, 60 min	1427 (@4 Bar, 22 °C)	30.0	27.0	[82]
PIM-1, UV in quartz, 5 min	3410 (@4 Bar, 22 °C)	20.7	18.5	[82]
PIM-1, UV in quartz, 60 min	1509 (@4 Bar, 22 °C)	27.2	28.6	[82]
Untreated PIM ref. [85]	4868 (@2 Bar, 35 °C)	-	15.4	[84]
O3-PIM-30	1033 (@2 Bar, 35 °C)	-	30.9	[84]
O3-PIM-60	443 (@2 Bar, 35 °C)	-	41.4	[84]

Notably, these TR membranes exhibited significantly improved mechanical properties compared to membranes derived from conventional polyimide precursors.

In 2013, Ma et al. studied pyrolyzed membranes derived from an intrinsically microporous polyimide with spirocenters (PIM-6FDA-OH). They used a step-wise heat treatment process at different temperatures between 440 and 800 °C [85]. At 440 °C, the PIM-6FDA-OH transformed into polybenzoxazole, resulting in a three-fold increase in CO_2_ permeability (from 251 to 683 Barrer) but a 50% reduction in selectivity over CH_4_ (from 28 to 14). At 530 °C, a unique amorphous carbon structure emerged, demonstrating superior gas separation properties. This included a 16-fold surge in CO_2_ permeability (4110 Barrer) and a significant 56% increase in CO_2_-probed surface area compared to the original polymer. The 600 °C heat produced a graphitic carbon membrane with impressive gas separation properties, including a 5040 Barrer CO_2_ permeability and a high CH_4_ selectivity of 38.

Recently, He et al. presented their study on creating high fractional free volume (FFV) endowed PIMs to achieve high gas permeability [86]. However, the overall selectivity was compromised. They deliberately employed an intermediate temperature range in an N_2_ atmosphere to fine-tune the microstructure of PIM-1 membranes, targeting improved gas separation performance. The combination of thermal-induced cross-linking and decomposition considerably elevated the micropores of PIM-1 membranes. This refinement significantly increased the membrane’s molecular sieving capability, leading to significant selectivity improvements: 350 for H_2_/N_2_, 1472 for H_2_/CH_4_, 3774 for H_2_/C_3_H_8_, and 197 for CO_2_/CH_4_. The H_2_ permeability reached 234 Barrer, surpassing the renowned “Robeson’s Upper Bound.”

In another study, Yerzhankyzy et al. reported on an intrinsically microporous hydroxyl-functionalized polyimide (PIM-PI) made of 4,4′-(hexafluoroisopropylidene)diphthalic anhydride (6FDA) and 2,6(7)-dihydroxy-3,7(6)-diaminotriptycene (DAT1-OH). This underwent thermal conversion to polybenzoxazole (PBO) [81]. This thermal rearrangement resulted in a significant increase in free volume, enhancing microporosity. The increase in free volume notably elevated gas permeability but also led to a reduction in gas-pair selectivity. The freshly produced PBO membrane (TRIP-TR-460-30), made with 460 °C thermal treatment for 30 min, achieved a 20-fold increase in CO_2_ permeability, reaching 840 Barrer from an initial 43 Barrer for the 6FDA-DAT1-OH polyimide. However, this came at the cost of an approximately 60% decrease in pure-gas CO_2_/CH_4_ selectivity, reducing from 52 to 21. The membrane also demonstrated favorable performance for propylene/propane separation and displayed mechanical properties similar to certain rigid polyimides, with a notable tensile strength.

#### 3.3.2. UV Treatment (Photo-Oxidative Modification)

Membranes can undergo photo-oxidative modification by exposing their surface to short-wavelength ultraviolet (UV) light in an oxygen-rich environment. This post-treatment alters the membrane’s structure and performance [87]. The duration of irradiation and the composition of the atmosphere are crucial for achieving specific surface modifications of the PIM membrane [82]. The reaction mechanism in question involves the homolytic cleavage to UV light of PIM-1. Within PIM-1, there are various potential sites for reaction, including =CH- in phenyl groups, >CH_2_, and −CH_3_ but the most probable site for reaction under UV irradiation is at the >CH_2_ group. Here, the covalent bonds are likely to break, releasing a hydrogen atom and leading to the formation of a radical-ion intermediate. Influenced by nearby alkyl groups, an intramolecular 1,2-migration reaction can occur, leading to the formation of a cyclohexyl radical intermediate. The reactive intermediate may then undergo further reactions, such as the abstraction of another hydrogen atom, leading to a more stable, rearranged PIM-1 structure with Spiro-Carbon Centre Destruction and Cyclohexyl Ring Formation [83].

In 2012, Li et al. demonstrated the potential of (UV)-rearranged PIM-1 membranes for H_2_/CO_2_ separation [83]. After 4 h of UV exposure, the PIM-1 membrane displayed an impressive 452 Barrer of H_2_ permeability along with a 7.3 H_2_/CO_2_ selectivity. Both experimental data and molecular simulation revealed that the polymer chains of PIM-1 underwent a 1,2-migration reaction, adopting a close-to-planar rearranged structure after UV radiation. The changes in PIM-1 post-UV treatment highlighted both chemical and structural transformations. The decrease in fractional free volume (FFV) and pore size primarily resulted from the disruption of the spiro-carbon center during UV radiation. Short UV exposure of PIM-1 of 5 and 10 min in the presence of O_2_ modified the membrane surface, leading to a reduced CO_2_ permeability but enhanced CO_2_/N_2_ and CO_2_/CH_4_ selectivity [82]. Even with the reduced CO_2_ permeability, the modified membranes still had a permeability two orders of magnitude higher than commercially available polymeric membranes. However, extending the exposure time from 20 to 40 min led to gains in CO_2_/N_2_ and CO_2_/CH_4_ selectivity but reduced CO_2_ permeability [82].

Recently, a study by Hou et al. proposed a dual method to boost membrane performance by integrating nanoparticle additives composed of a Porous Aromatic Framework (PAF-1) into a PIM matrix followed by UV irradiation [88]. Typically, crosslinking and nanoparticle incorporation lead to improvements in selectivity or permeability but often one comes at the other’s expense. In this study, the combination of nanoparticle PAF-1 and UV treatment resulted in a membrane with both remarkably improved membrane selectivity and high permeability. The H_2_/CH_4_ selectivity showed a 16-fold improvement, from 5.4 to 90, while the H_2_ permeability reached 4800 Barrer. This dual method allowed the PIM-1 MMM to outperform the 2015 upper bounds for H_2_/N_2_ and H_2_/CH_4_, as well as the 2008 upper bounds for H_2_/CO_2_ and CO_2_/CH_4_. Aging studies also showed that the combined modifications slowed down the physical aging rate compared to the unmodified PIM-1 membrane.

Recently, in situ ozone treatment of PIM-1 (O3-PIMs) has been introduced as an alternative oxidative modification method for H_2_ separation [84]. The ozone-treated PIM-1 displayed significantly higher selectivity for H_2_/N_2_, H_2_/CH_4_, and H_2_/CO_2_. After ozone oxidation, the O3−PIMs had reduced gas permeability but showed considerable increases in gas pair selectivity. This led to a notable enhancement in overall performance for H_2_/N_2_, H_2_/CH_4_, and H_2_/CO_2_, exceeding the latest benchmarks. For example, the O3−PIM-60 membrane exhibited a H_2_ permeability of 1294 Barrer and selectivity of 93.7, 121, and 2.92 for H_2_/N_2_, H_2_/CH_4_, and H_2_/CO_2_, respectively. The heightened selectivity in O3−PIMs is attributed to their enhanced size-sieving effect, resulting in better diffusion selectivity.

## 4. PIM-Based Mixed Matrix Membranes (MMMs) with Nanofillers

Over recent decades, there have been significant advancements in membrane-based CO_2_ capture and separation processes. A variety of membrane materials have been developed for this purpose, including polymeric membranes, Metal–Organic Framework (MOF) membranes, carbon molecular sieve membranes, and MMMs [41,89]. Among these materials, polymeric membranes have garnered significant attention due to their favorable attributes such as cost-effectiveness, processability, and mechanical strength. Nonetheless, polymeric membranes face a challenge known as the Robeson upper bound [15], wherein there exists a trade-off between permeability and selectivity. In contrast, inorganic membranes, particularly those composed of porous fillers, exhibit gas separation performances surpassing the Robeson upper bound. However, these inorganic membranes present difficulties in terms of complex preparation procedures, high cost, and brittleness compared to polymer membranes. Therefore, MMMs have emerged as a promising solution, capitalizing on the strengths of both inorganic materials and polymeric membranes. In MMMs, an organic polymer serves as the continuous matrix, while inorganic porous materials act as dispersed fillers. A variety of nanofillers, such as zeolites, silica, MOFs, Covalent Organic Frameworks (COFs), graphene/graphene oxide (GO), carbon nanotubes (CNTs), and carbon molecular sieves, have been employed as fillers in MMMs (see Table 3) [41].

**Table 3:** Performance of the PIM-based mixed matrix membrane with nanofillers for CO_2_ separation.

Inorganic Nanofillers	Membrane	CO_2_ Permeability (Barrer)	CO_2_/N_2_ Selectivity	CO_2/_CH_4_ Selectivity	Ref.
Silica Nanoparticles	PIM-1 ref. [90]	1250	19.8	-	[91]
	DMBA-NP (5–25% NPs)	2730–7930	16.8–20.5	-	[91]
Poly(ethylene glycol)-POSS (PEG-POSS)	PIM-1 ref. [92]	3795	12	19	[93]
	PIM-1/PEGPOSS (1–10)	1875–3381	18–31	13–30	[93]
Graphene Oxide (GO)	PIM-1 ref. [94]	6190		11.7	[90]
	ODAHGO-4 h (0.1–0.2%)	5429–6146	-	11.8	[90]
	P-H24 (1–10%)	5675–4727	-	12.2–12.6	[90]
Aged (850) days membrane	PIM-1 ref. [95]	2195	-	14.1	[92]
2D Reduced Holey Graphene Oxide	rHGO-TAPA (0.01–0.05–0.1)	2453–3245	-	13.2–15.9	[92]
(rHGO)	rHGO-tetrakis (0.01–0.05–0.1)	2738–3088	-	13.3–17.9	[92]
Organic Nanofillers					
Porous Aromatic Frameworks (PAFs)	PIM-1 ref. [96]	13,400	15	-	[94]
	PIM−SAP110	12,300	16	-	[94]
	PIM−SAP-OP	10,200	15	-	[94]
Porous Organic Frameworks (POFs)	PIM-1 ref. [97]	3694	18.9	-	[95]
	MAPDA/PIM-1-5/1-8/1-12/1-15/1-20	3048–7861	19.3–23.9	-	[95]
Covalent Triazine Framework (CTF)	PIM-1 ref. [98]	5800	-	11.5	[96]
Covalent Triazine Framework (CTF)	PIM-1@FCTF-1 (1–10%)	4700–9400	-	14.8–16.6	[96]
Beta-Cyclodextrin (β-CD)	PIM ref. [99]	3364	18.5	15.4	[96]
Beta-Cyclodextrin (β-CD)	PIM-CD-0.1/0.5/1/2%	3736–8812	18.3–25.4	12.3–17.2	[96]
Beta-Cyclodextrin (β-CD)	PIM-1/PDMS/PAN	402.6	21.3	-	[98]
Beta-Cyclodextrin (β-CD)	PIM-CD/PDMS/PAN	483.4	22.5–29.6	-	[98]
Metallic−organic Nanofillers					
Metal−Organic Frameworks (MOFs)	PIM-1 ref. [100]	274	-	3.44	[99]
	PIM-1/ZIF-7 (20)	218	-	5.29	[99]
	PIM-1/NH2-ZIF-7 (20)	249	-	6.06	[99]
Metal−Organic Frameworks (MOFs)	PIM-1 ref. [101]	7826	16.5	9.6	[102]
	NUS-8-NH2/PIM-1 (1.6/2.5/5.6/10.4/13.0/15.0%)	10,819–14,638	24.7–30.7	6.4–9.5	[102]

To achieve optimal performance, MMMs necessitate well-dispersed nanofillers and robust filler/polymer interfacial contact. In this context, we will discuss PIMs-based MMMs [101], highlighting their enhanced gas separation properties.

### 4.1. Inorganic Nanofillers

Incorporating inorganic nanofillers into PIM-based MMMs offers two main advantages: it boosts membranes’ CO_2_ separation properties and decreases their physical aging. This process disrupts the chain packing of PIMs, enhancing their longevity. This is crucial when choosing fillers to ensure a strong interfacial compatibility between the inorganic fillers and the polymer matrix. Enhancing this compatibility often involves functionalizing the surface of the filler.

Previous studies indicate that adding non-porous silica nanoparticles increased selective gas separation but reduced gas permeability [101,103]. However, recent research suggests that integrating these nanoparticles into glassy polymers enhances both gas permeability and selectivity [104,105].

**Figure 6:**
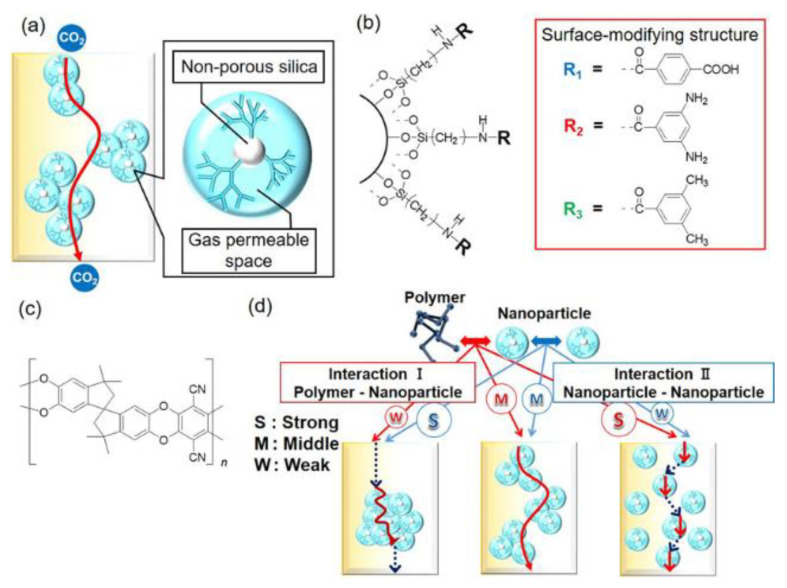
(**a**) Overview of the study’s concept: The MMM, a blend of PIM-1 and silica nanoparticles, featuring gas-permeable pathways. (**b**) Chemical modifications on the surface of silica nanoparticles. (**c**) Structure of PIM-1. (**d**) Illustration of diverse particle-assembled structures in MMMs, showcasing interactions between polymers and nanoparticles. Reprinted with permission from [91]. Copyright © 2023, American Chemical Society.

The Kawakami research group integrated PIM-1 with silica nanoparticles adorned with surface functional groups such as methyl, amine, and carboxylic acid moieties (Figure 6) [91]. This functionalization increased the interaction between the nanoparticle and the PIM-1 matrix, enhancing CO_2_ permeability and CO_2_/N_2_ selectivity in the MMM [90]. They utilized a variety of organic fractions, such as terephthalic acid (TPA), 3,5-diaminobenzoic acid (DABA), and 3,5-dimethylbenzoic acid (DMBA), for nanoparticle surface modification [91]. Unlike porous materials like zeolites and MOFs that boost gas permeability by introducing additional permeable routes, the dispersion of non-porous nanoparticles within the polymer matrix governs gas permeability and selectivity [91,104,105]. By integrating PIM-1 with modified silica nanoparticles, effective pathways are established through nanoparticle assembly, thereby influencing gas permeation dynamics across the PIM matrix (Figure 6) [91,104,105]. Nanoparticles with a strong affinity for the polymer disperse uniformly within the polymer matrix, preventing aggregate formation that could hinder gas diffusion pathways [91]. All MMMs with surface-modified silica nanoparticles exhibited superior performance, surpassing the benchmarks set by the 2008 Robeson upper bound compared to non-functionalized Si nanoparticles [91]. Especially noteworthy were the MMMs comprising PIM-1 and non-porous silica nanoparticles modified with a 25% weight load of DMBA-modified nanoparticles. These displayed an impressive CO_2_ permeability (7930 Barrer), a value six times greater than the original PIM-1 membrane (1250 Barrer) with only a minor 15% reduction in CO_2_/N_2_ selectivity (19.8 for the PIM-1 membrane and 16.8 for the MMM) [91]. Regarding physical aging, MMMs with DMBA-modified nanoparticles maintained elevated CO_2_ permeability without a drop in CO_2_/N_2_ selectivity after 60 days in standard conditions [91].

Silica derivatives, including pyrogenic silica and polyhedral oligomeric silsesquioxanes (POSS), are prevalent in PIM membranes for MMM fabrication [44,106,107]. Specifically, POSS stands out as an exceptional nanofiller for MMMs due to its nanometer-size porous structure (1–3 nm) and its ability to disperse uniformly in various polymers. Being an organic−inorganic hybrid material, it is ideal for crafting polymer−filler interfaces. Moreover, POSS structures provide a versatile platform for easy modification, facilitating the grafting of a broader range of functional groups and establishing multiple interaction channels [44,106,107]. The functionalization of POSS with various groups like alkyl, olefin, epoxy resin, alcohol, acid, amine, and sulfonate enhances filler dispersion in various polymer matrices such as PIM-1 and optimizes adsorption parameters for CO_2_ [44,106,107].

**Figure 7:**
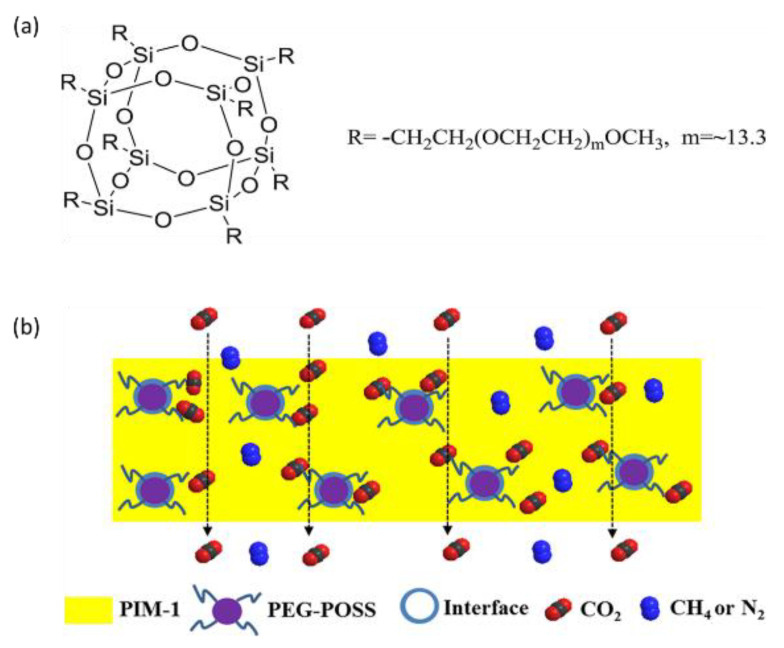
(**a**) Chemical structure of PEG−POSS; (**b**) MMMs PIM-1/PEG−POSS. Reprinted with permission from [93]. Copyright © 2023 Elsevier B.V. All rights reserved.

Jiang’s team [93] functionalized POSS nanofillers with poly(ethylene glycol) (PEG). These nanofillers can be incorporated into PIM-1, taking advantage of dipole−quadrupole interactions between the ethylene oxide units of PEG and CO_2_ molecules (Figure 7a). Their research revealed that while adding PEG directly to PIM-1 led to phase separation, covalently anchoring the PEG chains to POSS nanofillers led to homogeneously dispersed solutions [92]. Notably, the PIM-1/PEG-POSS membrane (Figure 7b) containing 10 wt% PEG−POSS nanoparticles exhibited optimal separation performance: CO_2_ permeability reached 1300 Barrer, with CO_2_/CH_4_ and CO_2_/N_2_ selectivity increasing by 150% and 63%, respectively, compared to pure PIM-1. This CO_2_/CH_4_ gas separation performance surpassed Robeson’s 2008 upper limit. Furthermore, PIM-1 showed reduced physical aging at high PEG−POSS concentrations. Overall, the developed PIM-1/PEG−POSS MMMs show great potential for CO_2_ capture [93]. Graphene oxide (GO) offers effective gas separation by forming molecular sieving galleries between adjacent nanosheets or through potential defects on these sheets [89]. Using GO as a filler has added advantages, as graphene is thought to influence chain packing and limit polymer chain mobility, potentially reducing membrane swelling and the process of physical aging [108].

The Liu team developed PIM-1/graphene oxide (GO) nanosheet MMMs. Uniformly assembled GO nanosheets in the PIM-1 matrix enhanced the gas separation performance of polymer membranes, forming diffusion channels that provide exceptional selectivity and permeability for CO_2_ capture [109]. The PIM-1/GO composite membrane was prepared using a standard solution casting and solvent evaporation method [109]. The resulting membrane showed GO nanosheets uniformly dispersed in the PIM-1, leading to a smooth surface and homogenous inner microstructure. Specifically, introducing GO nanosheets increases CO_2_ permeability from 3277 Barrer to 6169 Barrer while N_2_ permeability decreases from 230 Barrer to 50 Barrer. Compared with the PIM-1 membrane, the CO_2_ permeability value is doubled while the N_2_ permeability is four times lower. The GO nanosheets in the PIM-1/GO membrane enhance the membrane’s sieving ability, allowing for rapid transport of CO_2_ molecules while retaining N_2_ molecules. In addition, the CO_2_/N_2_ selectivity is 123.5, seven times higher than for the PIM-1-only membrane, indicating a significant improvement in the permeability and selectivity trade-off relationship.

Covalent functionalization of graphene surface is a strategy to create graphene-based polymer composites, enhancing the interfacial interaction between graphene and polymer matrices. Given its abundance of functional groups, including hydroxyl, epoxide, and carboxylic, GO offers numerous reactive sites for covalent functionalization. Gorgojo’s team prepared MMMs with PIM-1-functionalized GO derivates (3-aminopropyl)triethoxysilane (APTS−GO) [90]. Graphene oxide (GO) possesses hydroxyl, carboxyl, and carbonyl functional groups on its basal planes and edges, presenting active sites capable of interacting with aminopropyltriethoxysilane (APTS). Several membranes were fabricated with varying APTS−GO loadings spanning from 1 to 50 wt%. For gas separation performance assessment, a binary CO_2_ and CH_4_ mixture (50:50) was employed at 25 °C. The CO_2_ permeability of mixed PIM-1−APTS−GO membranes (denoted as P_A24) shows an initial minor reduction compared to the PIM-1-only membrane (6.190 Barrer). Remarkably, after 150 days, while the permeability of the PIM-1-only membranes declined, those with APTS−GO filler showed increased permeability. The most favorable results were achieved with a membrane containing 10 wt% of APTS−GO. This specific membrane preserved 85% of its initial CO_2_ permeability after 150 days, experiencing a mere drop of 310 Barrer. This drop signifies a nearly nine-fold reduction in CO_2_ permeability decline in comparison to the pure PIM-1 membrane. Concerning CO_2_/CH_4_ selectivity, the inclusion of PIM-1-functionalized filler enhanced the sieving capability of the membranes as compared to pure PIM-1 (approximately 11 for PIM-1 and 15 for PIM-1 + APTS−GO membranes). However, due to the notable variation in the average selectivity results, a clear optimal nanofiller loading yielding maximum selectivity was not discernible, as the values closely resembled those of pure PIM-1 membranes. These MMMs displayed a reduced extent of physical aging in comparison to their fully polymeric counterparts. This was manifested by a less pronounced reduction in both CO_2_ and CH_4_ permeability values over time.

Almansour et al. addressed the physical aging challenge by developing thin films of PIM-1 blended with 2D reduced holey graphene oxide (rHGO) nanosheets that were functionalized with amine groups [92]. By introducing rHGO nanosheets functionalized with tris(4-aminophenyl)amine at a filler content of 1 wt% into thin films of PIM-1, the CO_2_ permeance after 1 year of aging remained impressively close to its initial value. Notably, this value was twice as high as the CO_2_ permeance achieved by purely PIM-1 thin film composite membranes that had undergone a year of aging. Additionally, the aging behavior was further explored in membranes with thicknesses spanning several tens of micrometers (up to 2 years) and with a filler content of 0.1 wt%. The gas separation performance in these thicker membranes exhibited tendencies similar to those observed in thin films. This resulted in elevated CO_2_ permeability levels without compromising the CO_2_/CH_4_ selectivity.

Physical aging is a significant limitation for the industrial application of PIM-1 membranes. Documented by Luque-Alled et al. [110], this issue arises from the gradual reorganization of polymer chains after a PIM-1 film is formed, leading to a loss of free volume and, consequently, reduced gas permeability. This challenge is prevalent among glassy polymers with high free volume. To address this, researchers at the University of Manchester explored the integration of graphene-based nanofillers into PIM-1 as a countermeasure against physical aging. They experimented with various strategies, including the use of alkyl-functionalized graphene oxide (GO), reduced GO [111], organosilane-functionalized graphene oxide (APTS-GO), and GO functionalized with PIM-1 [90]. These graphene-based nanofillers, due to their ultrathin 2D structure, allowed for the creation of thin film membranes with thicknesses of only a few hundred nanometers. The study focused on the inclusion of holey GO nanofillers in PIM-1 membranes for CO_2_/CH_4_ gas separation. This was in response to the observed significant drop in gas permeability when non-holey graphene-like fillers were used, as previously reported [90,111]. The goal was to reduce physical aging while enhancing gas permeability. To achieve optimal dispersion within the polymer matrix, GO was functionalized with either octadecylamine or PIM-1 chains. The resulting MMMs were tested over 150 days, providing insights into the progression of physical aging and the influence of graphene-like nanofillers on gas permeability.

Finally, another alternative and cost-effective method to overcome physical aging has been identified in MMM systems that incorporate PIM-1 with clays. Here, PIM-1 polymer chains nestle within the clay’s interlayer spaces [94]. This confinement restricts chain movements, slowing the aging process. Gatti’s group highlighted this by using synthetic inorganic clays, known as saponites, as fillers to decelerate the physical aging of PIM-1 membranes [94]. They crafted MMMs from two distinct saponite variants—one purely inorganic and the other surfactant-functionalized. The performance of these membranes was monitored over a year to gauge the long-term effects of physical aging [94]. A year later, the native PIM-1’s CO_2_ permeability had plummeted by 80%. In contrast, the MMMs saw reductions of 53% and 59% for the inorganic and surfactant-functionalized saponites, respectively [94].

### 4.2. Organic Nanofillers

Organic fillers, including nonporous and porous organic nanomaterials, as well as polymers, have consistently shown superior interfacial compatibility with the PIM-1 matrix compared to inorganic nanofillers. This interaction can potentially modify the arrangement of PIM-1 chain segments. Furthermore, the incorporation of CO_2_-philic organic fillers might boost CO_2_ solubility within the membranes [100]. Organic nanomaterials, while insoluble, can be effectively dispersed in solvents, making them suitable fillers for PIMs. Beyond their superior compatibility with polymer matrices compared to inorganic counterparts, organic fillers can enhance CO_2_ selectivity and mitigate membrane aging. [112,113] Another promising solution to physical aging is the introduction of ultraporous additives. These additives can sustain the initial low-density porous stage of superglassy polymers. By absorbing a portion of the polymer chains inside their pores, they help maintain the chains in expanded configuration, preventing them from compacting during aging [112,113].

**Figure 8:**
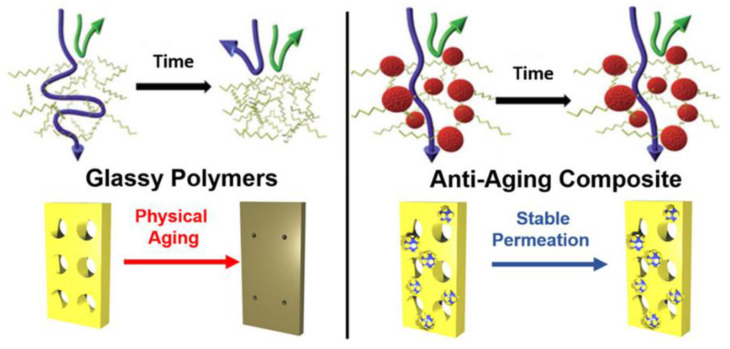
Schematic illustrations of a pure polymer membrane exhibiting packing of polymer chains post aging (**left**) and the PISA process that prevents chain relaxation, maintaining membrane permeability (**right**). Reprinted with permission from [113]. Copyright © 2023, American Chemical Society.

This phenomenon, known as the Porosity-Induced Sidechain Adsorption (PISA) phenomenon, combats aging in superglassy polymers. As a result, these polymers can sustain elevated CO_2_ permeability for up to a year and enhance CO_2_/N_2_ selectivity, as depicted in (Figure 8) [112,113]. This innovative strategy may pave the way for the broader commercial use of superglassy polymers in gas separation applications.

Ionic liquids (ILs) are emerging as a frontrunner among fillers for carbon capture membranes. Their appeal lies in their pronounced CO_2_ affinity, adaptable structure, and commendable compatibility and stability [114].

To understand the influence of ILs on the microstructure of polymer blends, PIM-1 was combined with various ILs, namely [C8MIM][Cl], [BMIM][DCa], [BMPyr][DCa], and [BMIM][Tf2N]. These were integrated using a casting method with chloroform as the solvent, resulting in polymer blends with up to 80 wt% IL content [115]. SEM images of the films showcase a variety of surface structures, heavily influenced by the type and concentration of the IL, especially evident with [BMIM][Tf2N] and [BMIM][DCa]. Serving as adaptable co-solvents, ILs enable the creation of controlled micropatterns in the polymer membranes. Different IL/PIM-1 weight ratios yield unique film structures. For instance, a lower IL concentration (<15 wt%) produces a dense film with uniform surface patterns, whereas a higher concentration (>50 wt%) leads to a porous film. This suggests that the film’s structure is a result of PIM-1’s limited solubility in ILs.

Inspired by the efficient CO_2_ transport in natural biological membranes, Wang et al. explored a blend of porous ionic polymers (PIPs) with filtration capabilities in MMMs [116]. These MMMs, based on PIM-1, were developed with three distinct pyridine-based PIPs (PIP-Py-X), each containing different anions like Cl^−^, Ac^−^, and BF_4_^−^. These PIP-Py-X polymers, known for their impressive CO_2_ adsorption capacity, integrate seamlessly with the PIM-1 matrix. The mobile anions in the MMMs serve as carriers, amplifying the movement of adsorbed CO_2_ through the membranes. Consequently, the PIM-Py-Ac-15 MMM showcases an outstanding CO_2_ permeability of 6205 Barrer and impressive CO_2_/N_2_ and CO_2_/CH_4_ selectivity ratios of 62.5 and 56.1, respectively.

In our recent work, we blended the ionic liquids [BMIM][Ac] and [BMIM][Succ] with PIM-1, aiming to combine PIM-1’s inherent high permeability with the high solubility of CO_2_ in the selected ILs [114,117,118]. Membranes with a PIM-1/[BMIM][Ac] ratio of 4:1 displayed almost twice the CO_2_ solubility at 0.8 bar, while other ratios maintained a CO_2_ solubility similar to pure PIM-1. The CO_2_ permeability of the blended PIM-1/[BMIM][Ac] membranes ranged from 1050 to 2090 Barrer for ratios of 2:1 and 10:1, respectively. Although these values are lower than those of pure PIM-1 membranes, they remained notably significantly permeable. Notably, these blend membranes, while having reduced permeability compared to PIM-1, exhibit increased CO_2_ solubility, leading to enhanced CO_2_/N_2_ selectivity.

Porous Aromatic Frameworks (PAFs) stand out as valuable porous additives, especially for their expansive surface area (>5000 m^2^ g^−1^) and remarkable chemical and physical stability. These attributes make PAFs ideal for integration into mixed matrices with superglassy polymers [112,113]. Portions of PIM-1 delve into PAF’s pores, creating a cohesive nanocomposite that restricts polymer chain movements and conserves small free volumes. This structure not only mitigates aging effects but also boosts both permeability and selectivity [112,113]. When evaluating the permeability values for CO_2_ and CO_2_/N_2_ selectivity across various nanocomposites derived from superglassy polymers (like PTMSP, PMP, and PIM-1) against Robeson’s Upper Bound [112], a clear distinction emerges. PIM-1/PAF-1 films outperform both pure glassy polymer films and those combined with metal−organic compounds, surpassing Robeson’s 2008 limit [112]. However, while PAF-1 exhibits the most potent anti-aging properties among the porous additives studied, its high production cost poses a barrier to industrial adoption [94,112,113].

Covalent Organic Frameworks (COFs) [96,119], Porous Organic Frameworks (POFs) [95], and Porous Organic Polymers (POPs) [120] have also been identified as potent organic fillers for crafting MMMs based on PIM.

COFs, in particular, have been proposed as additives in polymeric membranes for CO_2_ separation [121,122]. Researchers have highlighted the synergy between PIMs and COFs, demonstrating the potential of these organic fillers to amplify the gas separation capabilities of such membranes. Wu et al. delved into the application of a PIM/COF MMM in gas separation [119]. They found that the integration of COF (SNW-1), recognized for its optimal porosity and compatibility with the PIM-1 matrix, markedly improved CO_2_ diffusion within the membrane. Compared to the pure PIM-1 membrane, the formulated MMMs showcased a 27.4% and 37.6% surge in CO_2_/CH_4_ and CO_2_/N_2_ selectivity, respectively. This was accompanied by a significant 116% increase in CO_2_ permeability. Jiang et al. incorporated a CO_2_-philic perfluorinated covalent triazine framework (FCTF-1) into a PIM-1 matrix [96]. This aimed to simultaneously bolster gas selectivity and permeability. The organic nature of FCTF-1, which combines its polar elements like triazine rings and fluorine atoms, favored CO_2_ adsorption over CH_4_, enhancing solubility selectivity. Moreover, the microporosity of FCTF-1 further refined diffusion selectivity. Employing PIM-1@FCTF-1 MMMs with 2 wt% filler, CO_2_ permeability reached 7300 Barrer, with a CO_2_/CH_4_ selectivity of 16.6. Preliminary results also hint at the potential PIM-1@FCTF-1 MMMs in other small molecule separation contexts, such as propene/propane.

Yu et al. introduced a unique nanofiller class called MAPDA, a type of POF fabricated using a triangular melamine (MA) monomer and a linear 1,4-piperazinedicarboxaldehyde (PDA) monomer [95]. Adsorption tests revealed MAPDA’s impressive CO_2_ adsorption capabilities, registering an uptake of 47.0 cm^3^ g^−1^ at 298 K and 101 kPa. Incorporating MAPDA into PIM-1 matrices led to MMMs with varied MAPDA concentrations (0–20 wt%). Gas permeation tests showed a marked rise in CO_2_ permeability, jumping from 3694.5 to 7861.9 Barrer. Concurrently, CO_2_/N_2_ selectivity climbed from 18.9 to 23.9 when juxtaposing pure PIM-1 against a MAPDA/PIM-1 membrane with 15 wt% MAPDA. This boost can be credited to MAPDA’s inherent high porosity and molecular affinity.

Selecting the right fillers is essential, especially when considering the compatibility between the filler and the polymer. POPs stand out as a promising choice for organic fillers in formulating PIM-1-based MMMs for gas separation. Wang et al. highlighted a tryptone-based POP (TPFC) with two distinct amine modifications (TPFC-CH_2_NH_2_ and TPFC-CH_2_PEI) as fillers [120]. The notable CO_2_ adsorption capacity of these additives led to significant enhancements in both CO_2_ permeability and selectivity for the developed MMMs, surpassing Robeson’s 2008 benchmark for CO_2_/N_2_ and CO_2_/CH_4_ gas pairs. Specifically, the PIM−TPFC−CH_2_NH_2_ showcased a CO_2_ permeability of 7730 Barrer, with CO_2_/N_2_ and CO_2_/CH_4_ selectivities of 45.9 and 36.4, respectively.

**Figure 9:**
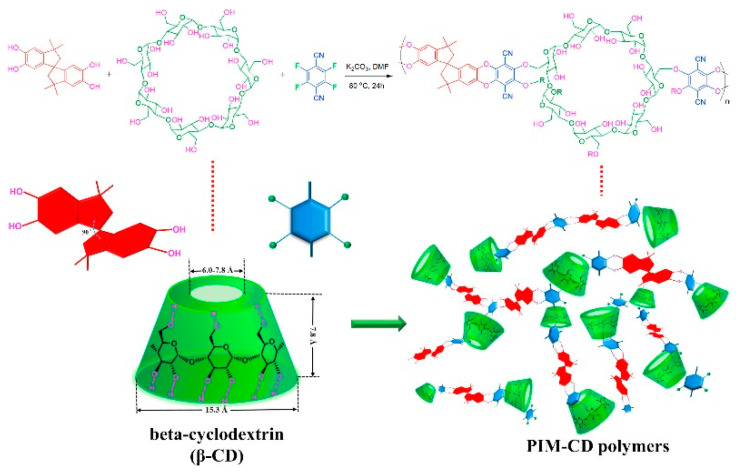
Synthesis of the PIM and PIM−CD polymers via aromatic nucleophilic substitution polymerization of β-CD, TTSBI, and TFTPN. Reprinted with permission from [97]. Copyright © 2023 Elsevier B.V. All rights reserved.

Beta-cyclodextrin (β-CD), made up of seven α-D-glucose units arranged in a ring, stands out as a notable organic additive in the market. The structure of β-CD resembles a distinctive 3D hollow bowl, boasting an internal cavity diameter ranging from 6.0 to 7.8 Å [97,98]. Various techniques have been explored to integrate beta-cyclodextrin into polymer matrices. A straightforward physical mixing approach often faces issues of phase separation, primarily due to the agglomeration of CD particles, especially at high CD loadings [97]. One effective strategy to mitigate these challenges is to incorporate β-CD directly into the polymer chain using reactive chemical bonds. This not only enhances phase separation properties but also prevents the reduction of free volume in PIM materials. Consequently, numerous methods have been devised to functionalize the hydroxyl groups of β-CD [97]. These techniques produce β-CD variants equipped with reactive groups, which can then chemically bond with the functional groups of monomers, ensuring a robust connection between β-CD and the polymer [97,98] (Figure 9).

Incorporating β-CD, which possesses multi-reactive hydroxyl groups, into PIM achieves multiple benefits. It not only introduces additional free volume essential for gas transport but also induces a mildly crosslinked structure, fortifying the polymer matrix. The synergistic interactions between β-CD and PIM serve to limit chain mobility and hinder micropore collapse. As a result, PIM−CD membranes display a markedly enhanced resistance to physical aging in comparison to their PIM counterparts. The Chung team has delved into the gas separation efficacy and physical aging characteristics of PIM−CDs. Notably, as β-CD loading in PIM−CD membranes increases, there is a corresponding rise in permeability compared to unmodified PIM membranes Pure gas permeation tests underscore that even a minimal addition of β-CD to the PIM structure can amplify CO_2_ permeability from 3368 to 8812 Barrer (tested at 20 bar and 35 °C)—a substantial 162% surge—without a significant compromise on gas selectivity. Impressively, PIM−CD membranes outperform the 2008 Robeson Upper Bound for nearly all pure gases [97,98,100]. When evaluating the physical aging of both PIM and PIM−CD membranes, the latter consistently showcases superior aging resistance. This is likely attributed to the β-CD units anchoring the polymer chains via ether bonds. This anchoring preserves the structural integrity, rendering the polymer chains exceptionally rigid and thereby preventing potential collapse [97,98,100]. Given that β-CD is a readily accessible organic material in the commercial market, PIM−CD membranes emerge as promising contenders for natural gas separation. Their potential for scalability also makes them attractive for broader industrial applications.

### 4.3. Metallic-Organic Nanofillers

Metal−Organic Frameworks (MOFs) represent a cutting-edge evolution in fillers, standing out against traditional inorganic nanoporous fillers like zeolite, activated carbon, and silica. The hallmark of MOFs lies in their meticulously structured pores and customizable organic ligands, positioning them as elite fillers in the domain. While MOF membranes [123], alongside zeolite- [124] and carbon-based membranes [125], are employed in gas separation for their impressive selectivity and permeability, MOFs’ mass production remains a challenge. In contrast, polymeric membranes are favored industrially for their unparalleled scalability and robust chemical−physical resilience.

To enhance PIMs’ gas separation attributes and navigate the permeability/selectivity conundrum, numerous studies have delved into MOF−PIM membranes, addressing applications from biogas enhancement to CO_2_ capture [99]. Integrating a MOF subclass with zeolite-like structures, known as Zeolitic Imidazolate Frameworks (ZIFs), including variants like ZIF-8 [126,127], ZIF-67, and ZIF-71 [128] into the rigid polymer PIM-1 often boosts gas permeability. However, this does not significantly elevate selectivity. The inherent rigidity of glassy PIM-1 complicates effective bonding with fillers, leading to the emergence of interfacial gaps that diminish selectivity [99]. To address this, various strategies have been pursued. These encompass the use of specialized organic binders with diverse functional groups, refining filler geometry, and modifying the filler’s surface either chemically or physically to bolster its adherence to the polymer matrix. Another tactic entails tailoring the interaction between the filler and polymer matrix, aiming for a homogenous filler distribution and ensuring harmonious compatibility between the two components [99,100].

Wang et al. sought to address the aforementioned limitation by introducing an amino group to ZIF-7 [99]. This modification of ZIF-7 not only showcased remarkable intrinsic CO_2_/CH_4_ separation capabilities but also fostered a beneficial interaction with the PIM-1 polymer. Furthermore, the resistance to physical aging was enhanced: after 120 days, the PIM-1/NH_2_−ZIF-7 membrane experienced only a 26% decline in permeability, in contrast to the pure PIM-1 membranes, which saw a 50% reduction in CO_2_ permeability under identical conditions. The inclusion of NH_2_−ZIF-7 in these membranes promotes the formation of interfacial hydrogen bonds, which likely restrict the movement of polymer chains, thereby mitigating aging effects. In the same year, Wang et al. highlighted a ZIF-embedded PIM-1 that exhibited heightened CO_2_/CH_4_ selectivity, crucial for biogas enhancement. Specifically, the selectivity of PIM-1/NH_2_−ZIF-7 surged by 75.6% when compared to the unmodified PIM-1 membrane [99].

The NH_2_−ZIF-7 not only excels in CO_2_/CH_4_ separation but also synergizes well with the PIM-1 polymer. The presence of NH_2_ augments the polymer/ZIF interaction, leading to polymer chain rigidity and the partial encapsulation of NH_2_−ZIF-7 particles. More recent studies have highlighted the use of amino-functionalized MOF nanosheets (NUS-8−NH_2_) integrated into PIM-1 for CO_2_ separation [102]. Incorporating amino groups into NUS-8−NH_2_/PIM-1 MMMs achieves two primary objectives: it ensures a uniform dispersion of MOF nanosheets, creating swift transport channels within the layers, and it bolsters CO_2_ transport. These MMMs can accommodate a high filler content of up to 15 wt% without undermining their separation efficacy, thanks to the hydrogen bonding between the NUS-8−NH_2_ filler and the PIM-1 matrix. Specifically, an MMM with 10 wt% NUS-8−NH_2_ showcases a CO_2_ permeability nearing 14,000 Barrer and a CO_2_/N_2_ selectivity around 30, outperforming Robeson’s 2008 benchmark.

### 4.4. Multiple Fillers

The pursuit of efficient gas separation technologies has led to the exploration of MMMs that synergistically combine the benefits of both organic and inorganic fillers. By integrating diverse fillers into a single membrane, researchers aim to harness the unique properties of each component, resulting in enhanced performance and durability. This approach not only optimizes the separation capabilities but also addresses challenges like physical aging, ensuring long-term stability and effectiveness.

Examples of this innovative approach have been documented, particularly in the context of PIM. One notable instance involves the incorporation of a task-specific ionic liquid (TSIL) into TSIL@ZIF-67/PIM-1 MMMs. This combination capitalizes on the high CO_2_ solubility of TSIL and its exceptional compatibility with both ZIF-67 and PIM-1 [129]. With just 10 wt% of TMGHIM@ZIF-67, the resulting MMMs showcased remarkable enhancements in CO_2_/N_2_ and CO_2_/CH_4_ selectivities, achieving values of 13.7 and 10.5, respectively. Furthermore, CO_2_ permeability soared to an impressive 12,848.5 Barrer, marking a 2.5-fold increase compared to pure PIM-1 membranes. A standout feature of these MMMs is their robust resistance to physical aging, ensuring longevity and consistent performance.

Sabetghadam et al. demonstrated another synergistic approach, where the integration of glassy Matrimid polymeric chains with high-performance PIM-1 led to the creation of MMMs. These membranes, fortified with MOF fillers like NH2−MIL-53(Al), exhibited exceptional separation capabilities and showcased minimal degradation even in humid conditions. Impressively, their efficacy in post-combustion CO_2_ capture remained undiminished even after 17 months of aging [130].

Further advancements in this domain include the development of MMMs tailored for post-combustion CO_2_ capture and biogas upgrading. These membranes, derived from PIM-1 (10–90 wt%)/6FDA-DAM blends and fortified with ZIF-8 filler (3–20 wt%), underwent evaluations for CO_2_/CH_4_ and CO_2_/N_2_ separations [131]. The findings revealed that increasing the proportion of PIM-1 in the blend amplified gas permeabilities. However, there was a trade-off as selectivity for both gas mixtures diminished. Yet, the introduction of even modest amounts of ZIF-8 into these MMMs significantly boosted their gas separation ability. Among the tested compositions, blends containing a PIM-1/6FDA-DAM weight ratio of 10/90, complemented with 10 wt% of ZIF-8, stood out. These membranes delivered a CO_2_ permeability of 2891 Barrer, a CO_2_/CH_4_ selectivity of 26.6, and a CO_2_/N_2_ selectivity of 18.1, underscoring their potential in efficient gas separation applications.

## 5. Conclusions

PIMs’ inherent microporosity and high permeability have positioned them as one of the favorite polymers to develop efficient gas separation materials. Their unique structural attributes facilitate selective gas transport, making them indispensable for critical applications, from carbon capture to hydrogen purification. But what truly amplifies the potential of PIMs is their synergy with a plethora of fillers. We have delved into several types of fillers such as Metal−Organic Frameworks (MOFs), zeolites, and even graphene oxide. Each of these fillers, with their distinct characteristics, interlinks with PIMs in unique ways, enhancing the separation performance of the resultant membranes. Besides this strategy, post-modification of PIMs and the creation of novel PIMs have been demonstrated to be winning strategies.

However, this field is not without its challenges. While the potential of PIMs and their composite membranes is vast, we must also deal with issues like physical aging, the intricacies of filler dispersion, and the ever-present question of long-term stability. These challenges beckon the scientific community to delve deeper, to experiment, and to innovate. Looking ahead, there is a vast expanse of uncharted territory. The long-term performance of these membranes, especially when subjected to rigorous industrial conditions, remains a question mark. Moreover, the world of fillers is vast and varied, and the potential for discovering novel fillers that could revolutionize PIM-based membranes is immense.

In conclusion, as we stand at the crossroads of sustainability and technological advancement, the exploration of PIMs and various fillers offers a beacon of hope. The progress we make in this domain will undoubtedly shape the future of gas separation technologies, directing us towards a more sustainable and energy-efficient world.

## Figures and Tables

**Table 1:** Permeation properties of commercial polymers for CO_2_ separation.

Polymer	Tg of Polyether Block (°C)	Tm of Polyether Block (°C)	CO_2_ Permeability (Barrer)	CO_2_/N_2_ Selectivity	Ref.
PEBAX 4011	−53	9	66	56.4	[31]
PEBAX 1074	−55	−1	120	51.4	
PEBAX 4033	−78	11	113	20.4	
PEBAX 2533	−77	13	221	23.4	
PEO1000-T6T6T	−45	−2	75	41	[32]
PEO2000-T6T6T	−48	21	180	49	
(PEO600/T)2500-T6T6T	−44	−6	121	50	
(PEO600/T)5000-T6T6T	−43	−3	174	53	

## Data Availability

Not applicable.
